# Dysregulated NAD(H) homeostasis associated with ciprofloxacin tolerance in *Escherichia coli* investigated on a single-cell level with the Peredox [NADH:NAD+] biosensor

**DOI:** 10.3389/fmicb.2023.1191968

**Published:** 2023-06-21

**Authors:** Joanna Urbaniec, Martina Sanderson-Smith, Johnjoe McFadden, Faisal I. Hai, Suzanne M. Hingley-Wilson

**Affiliations:** ^1^Department of Microbial Sciences, University of Surrey, Guildford, United Kingdom; ^2^School of Civil, Mining and Environmental Engineering, University of Wollongong, Wollongong, NSW, Australia; ^3^School of Chemistry and Molecular Bioscience, University of Wollongong, Wollongong, NSW, Australia

**Keywords:** *Escherichia coli*, tolerance, AMR, antibiotic, ciprofloxacin, NADH, single-cell, biosensor

## Abstract

**Introduction:**

Antibiotic persistence (subpopulation tolerance) occurs when a subpopulation of antibiotic sensitive cells survives prolonged exposure to a bactericidal concentration of an antibiotic, and is capable of regrowth once the antibiotic is removed. This phenomenon has been shown to contribute to prolonged treatment duration, infection recurrence, and accelerated development of genetic resistance. Currently, there are no biomarkers which would allow for segregation of these antibiotic-tolerant cells from the bulk population prior to antibiotic exposure, limiting research on this phenomenon to retrograde analyses. However, it has been previously shown that persisters often have a dysregulated intracellular redox homeostasis, warranting its investigation as a potential marker for antibiotic tolerance. Furthermore, it is currently unknown whether another antibiotic tolerant subpopulation - viable but non-culturable cells (VBNCs), are simply persisters with extreme lag phase, or are formed through separate pathways. VBNCs similarly to persisters remain viable following antibiotic exposure, however, are not capable of regrowth in standard conditions.

**Methods:**

In this article we employed an NADH:NAD+ biosensor (Peredox) to investigate NADH homeostasis of ciprofloxacin-tolerant *E. coli* cells on a single-cell level. [NADH:NAD+] was used as a proxy for measuring intracellular redox homeostasis and respiration rate.

**Results and Discussion:**

First, we demonstrated that ciprofloxacin exposure results in a high number of VBNCs, several orders of magnitude higher than persisters. However, we found no correlation in the frequencies of persister and VBNC subpopulations. Ciprofloxacin-tolerant cells (persisters & VBNCs) were actively undergoing respiration, although at a significantly lower rate on average when compared to the bulk population. We also noted significant heterogeneity on a single-cell level within the subpopulations, however were unable to segregate persisters from VBNCs based on these observations alone. Finally, we showed that in the highly-persistent strain of *E. coli, E. coli* HipQ, ciprofloxacin-tolerant cells have a significantly lower [NADH:NAD+] ratio than tolerant cells of its parental strain, providing further link between disturbed NADH homeostasis and antibiotic tolerance.

## 1. Introduction

Cellular redox environment can be broadly defined as the balance between oxidants: reactive oxygen species (ROS), reactive nitrogen species (NOS) and hydrogen peroxide, and antioxidants – ROS/NOS-degrading enzymes, e.g., superoxide dismutase which breaks down superoxide ions (Reniere, 2018). During infection, ROS produced by phagocytic cells have been shown to contribute to pathogen clearance (Li et al., 2021). Furthermore, bactericidal action of multiple antibiotic classes has been linked to ROS-mediated damage (Kohanski et al., 2007; Dwyer et al., 2014). Therefore, it is reasonable to assume that differential regulation of redox pathways would also be involved in persistence/tolerance to antibiotics, with antibiotic-tolerant cells being able to ‘evade’ damaging action of antibiotic and/or phagocyte-generated ROS as a result of their transient metabolic state. Indeed, it has recently been demonstrated that reducing reductase activity of ampicillin and ofloxacin persisters with chlorpromazine (CPZ), an FDA approved antipsychotic drug, resulted in 1000-fold reduction of the persister fraction (Mohiuddin et al., 2020a), suggesting that compounds affecting bacterial reductase activity could be effective as anti-persister drugs. However, it should be noted that many commercial kits measuring reductase activity do not disclose the specifics of assay components or reactions due to patenting rights, which can make it difficult to determine specific enzyme(s) whose activity is altered in the antibiotic-tolerant state.

Alternatively, NADH:NAD+ ratio detection system can be used as a proxy to estimate the intracellular redox homeostasis. NAD(H) acts as a co-factor in hundreds of intracellular redox reactions, including oxidative respiration (a major ROS-producing pathway) (Massudi et al., 2012), warranting further investigation of its use as a biomarker for antibiotic tolerance. Furthermore, intracellular [NADH:NAD+] strongly correlates with the cell’s respiration rate (Pinzon et al., 2011; Ricciardi et al., 2014; Braissant et al., 2020). A fluorescent biosensor, where a sensor protein is designed to fluoresce upon NADH binding can be used to quantify intracellular NAD(H). The first such biosensor, Peredox, was developed by Hung et al. (2011) and consists of a circularly permutated & pH-stable T-Sapphire protein, flanked by NADH-binding protein Rex. Upon binding of NADH, the biosensor changes its conformation, resulting in concentration-dependent increase in T-Sapphire fluorescence. Peredox can also bind NAD+ (although at lower affinity), without increase in fluorescence, and therefore this biosensor reports the relative cytosolic NADH:NAD+ ratio (Hung et al., 2011).

In this study we employed the Peredox biosensor to measure cytosolic [NADH:NAD+] of the highly persistent *E. coli* strain: *E. coli* HipQ (Wolfson et al., 1990; Hingley-Wilson et al., 2020), and its parental strain, prior, and post exposure to, high concentrations of ciprofloxacin. We present first-time observations of NADH homeostasis of antibiotic-tolerant and antibiotic susceptible cells, on a single cell level. Furthermore, we demonstrate that a lower [NADH:NAD+], i.e., a lower cellular respiration rate relates to ciprofloxacin tolerance.

## 2. Materials and methods

Please refer to supplementary material for Peredox biosensor plasmid map (Supplementary Figure S1).

*E. coli* HipQ and its parental strain were a kind gift from Nathalie Q. Balaban. The strain was first described by Wolfson et al. (1990).

### 2.1. Peredox biosensor transformation into *E. coli*

Overnight *E. coli* cultures were diluted 1:200 in super optimal broth (SOB) (*Sigma Aldrich*) and incubated at 37°C until an OD_600_ value of 0.3–0.5 (~3 h). Next, the cultures were spun at 5000 g for 10 min. The supernatant was removed and remaining pellet was concentrated 10 times in transport and storage solution (TSS: LB Miller supplemented with 10% PEG 3350, 20 mM MgSO_4_ and 5% dimethyl sulfoxide), placed immediately on ice and left for an hour. 100–200 ng of DNA to be transformed was diluted to 200 μL in chilled TCM (10 mM Tris/HCl at pH 7.5, 10 mM MgCl_2_,10 mM CaCl_2_) and kept on ice. 200 μL of prepared as above competent cells was then transferred to tubes containing the TCM-diluted DNA and mixed by pipetting, taking care to not warm up the tubes. The mix was left on ice for an hour. Afterwards the samples were transferred to a heating block set to 45°C for 2 min and returned to ice for 5 min. Next the samples were diluted with 0.6 mL of SOC (SOB medium supplemented with 20 mM filter-sterilized glucose) and incubated at 37°C, with shaking at 200 rpm for 1 hour. 100 μL of the sample was then spread on Lysogeny Broth (LB) plates supplemented with 100 μg/mL ampicillin and incubated at for 24 h. PUC19 plasmid was used as positive transformation control and non-transformed competent *E. coli* cells were used as the negative control.

### 2.2. Peredox expression assay

Glycerol (25%) in LB bacterial stocks were stored at −80°C, streaked on LB (*Sigma Aldrich*) agar plates or LB agar plates containing 100 μg/mL of ampicillin for pRSETB-Peredox transformed strains. Pates were incubated at 37°C overnight. Next, single colonies were inoculated into 1 mL of LB or LB with 100 μg/mL ampicillin added for plasmid retention. Cultures were incubated at 37°C overnight, with shaking at 200 rpm. Overnight cultures were then transferred to a black, clear-bottom, 96-well plate (*ThermoFisher Scientific*) (200 μL/well). Total biomass (OD_600_) and fluorescence intensity measurements were performed in the CLARIOstar plate reader (*BMG LabTech*). Fluorescence intensity was recorded at 575 nm excitation, 610 nm emission for mCherry (plasmid copy number), in precise mode, sequential well scan and automatic gain adjustment. Fluorescence intensity was normalized to OD values and compared between pRSETB-Peredox-transformed and untransformed samples in GraphPad Prism *(GraphPad).*

### 2.3. Time-kill assays

Overnight (16–18 h) *E. coli* cultures were diluted to an OD_600_ of 0.05 in LB and incubated for 2–3 h at 37°C, with shaking at 200 rpm until the cultures reached mid-log phase of growth (OD_600_ of ~1 or CFU/mL of 8^10^8^–1.5^10^9^). OD_600_ measurements in a 1 cm plastic cuvette, as well as a Miles & Misra (MM) dilution series (Miles et al., 1938) on LB agar plates was performed to determine starting CFU/mL values (i.e., time 0, prior to antibiotic addition). MM dilutions were prepared as 200 μL final volume and 10 μL/‘spot’ were plated with 3 ‘spots’ per replicate Next, ciprofloxacin was added at 25XMIC concentration (0.4 μg/mL, determined experimentally) to achieve concentration-independent killing, as outlined by a recent consensus statement (Balaban et al., 2019). Cultures were then returned to the shaking incubator and an MM series was performed at 5 and 24 h following antibiotic addition. At T_5_ and T_24_ antibiotic was removed by washing prior to carrying out the MM series: 100 μL sample was removed from the culture at each timepoint and centrifuged at 5000 *g* for 5 min. The cell pellet was then resuspended in 100 μL of standard phosphate-buffered saline (PBS), pH 7.4 *(Sigma Aldrich)* or fresh media and this suspension was used in the MM dilution series. MM plates were incubated at 37°C for 48 h, as it was previously determined that the HipQ strain persisters required 48 h for regrowth on agar plates. For direct comparison, time-kill assays and cytosolic [NAD(H)] (section 2.4) measurements were carried out on the same bacterial cultures simultaneously.

### 2.4. Cytosolic [NAD(H)] measurements with flow cytometry

The time-kill assay method described above was followed, with the exception that at T_0_ (prior to antibiotic addition) and T_24_ (24 h post antibiotic addition) sample aliquots were diluted 1:500 in sterile PBS (to 10^6^ total cells/mL). Next, cells were live/dead stained with Zombie near-infrared (near-IR) fixable dye (*BioLegend*) (1:1000 or 1 μL/10^6^ cells) and incubated for 20 min in the dark. For heat-killed control samples, undiluted aliquots (1 mL) were incubated at 75°C for 30 min and diluted to 10^6^ total cells/mL in PBS prior to live/dead staining. Next, cells were loaded onto the LSR Fortessa Cell Analyser *(BD Biosciences)* and forward scatter (FSC), side scatter (SSC) and zombie near-IR fluorescence (650 nm excitation, 746 nm emission) was recorded. Additionally, T-sapphire fluorescence intensity ([NADH:NAD+]) was recorded at 405 nm excitation and 520 nm emission and mCherry fluorescence intensity (Peredox plasmid copy number) was recorded at 561 nm excitation and 610 nm emission wavelengths. Laser power was kept consistent throughout the independent experiments. Subsequent data analysis was performed in FlowJo v.10.8 *(BD Biosciences).* First, doublets were excluded based on FSC-A/FSC-H ratio (Cosma, 2020) and then cell population was gated manually based on FSC & SSC values. Live/dead discrimination was performed by applying a bifur gate on histogram plot of zombie near-IR fluorescence, using live cells (mid-exponential cultures, no antibiotic) and heat-killed cells as negative and positive staining controls, respectively. The singlet subpopulation was further split into NADH biosensor expressing (mCherry positive) and non-expressing subpopulations (mCherry negative) with a bifur gate, using untransformed cells as the negative control. Untransformed cells were also used to quantify autofluorescence for both aforementioned fluorophores. To quantify relative NAD(H) of individual cells, T-sapphire fluorescence intensity was plotted against mCherry fluorescence intensity of all events was done in FlowJo v.10.8 *(BD Biosciences).* For statistical analysis, fluorescence intensity values of T-sapphire and mCherry of individual events were exported into Microsoft excel, T-sapphire fluorescence was normalized to mCherry fluorescence and values were averaged within biological replicates. All statistical analyses (see respective figure legends) were performed in GraphPad Prism v.8. The antibiotic tolerant subpopulation (persisters & VBNCs) was classified as cells which stained as ‘live’ following antibiotic exposure was estimated by subtracting the % of persister cells in a given biological replicate from the % of live cells at T_24_.

## 3. Results

### 3.1. pRSETB-Peredox ([NADH:NAD+] biosensor) is expressed in *Escherichia coli* in the absence of T7 polymerase

pRSETB-Peredox (also referred to as NADH biosensor) was successfully transformed into the highly persistent *E. coli* HipQ and its parental strain, confirmed by growth on ampicillin (pRSETB selection marker) agar plates. The biosensor consists of T-Sapphire-Rex fusion protein whose fluorescence intensity correlates with intracellular [NADH:NAD+], as well as constitutively expressed mCherry which can be used to normalize T-sapphire fluorescence to plasmid copy number (Hung et al., 2011; see Supplementary Figure S1 for pRSETB-Peredox plasmid map).

**Figure 1:**
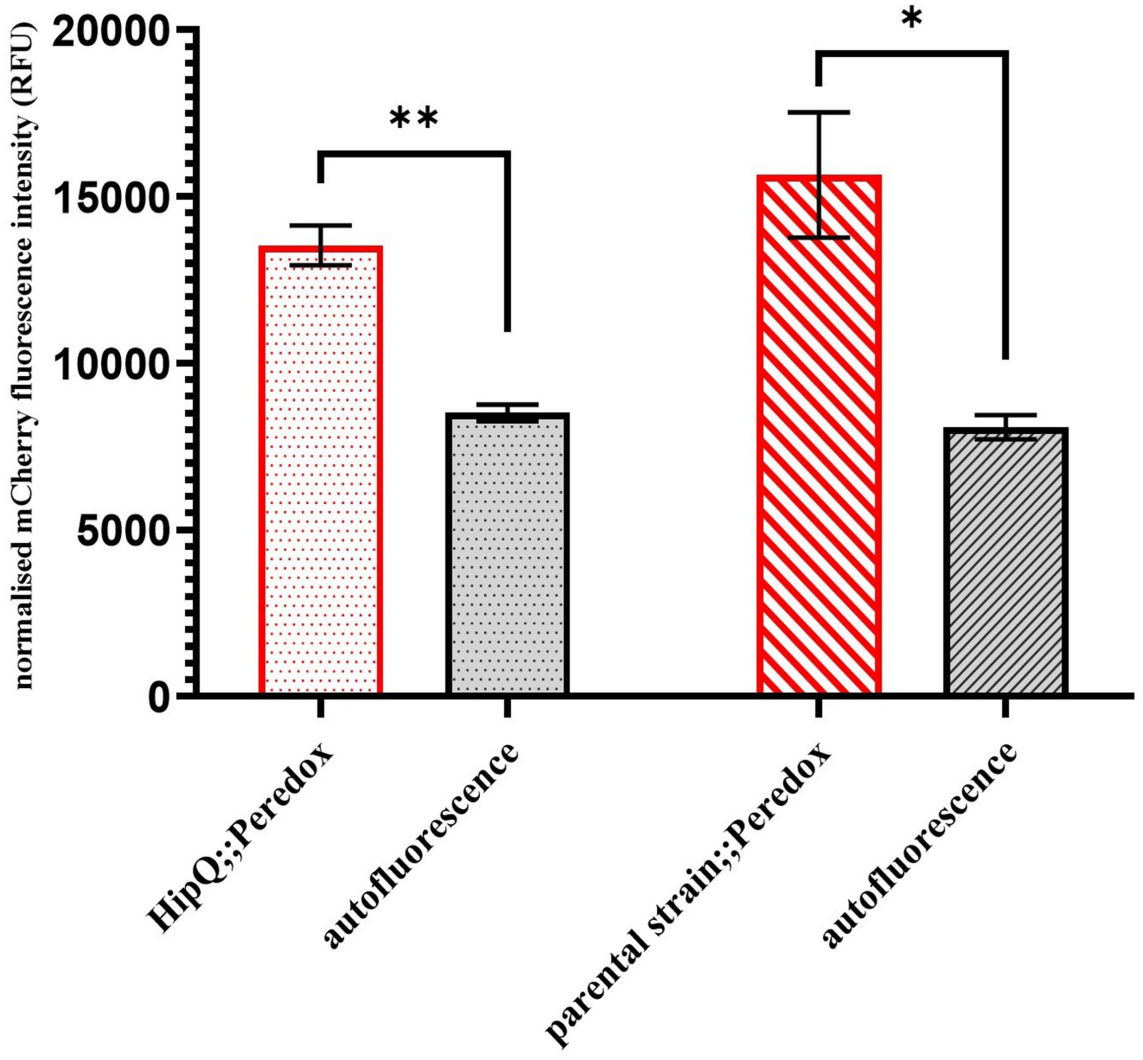
pRSETB-Peredox ([NADH:NAD+]) biosensor is expressed in *E. coli* even in the absence of the T7 polymerase expression. mCherry fluorescence (biosensor copy number) was measured at 575nm excitation, 610nm emission (575/610). Autofluorescence is based on fluorescence intensity of untransformed cultures and fluorescence intensity was normalised to sample biomass. n-3 biological replicates, error bars are SEM, * *p <* 0.05, ** *p* < 0.01 and q < 0.05, by repeated measures ANOVA with Geisser-Greenhouse correction and Benjamini, Kreuger and Yekutieli false discovery control method.

Since NADH biosensor expression is driven by the viral T7 promoter (Supplementary Figure S1), initially no expression was expected without a subsequent transformation of *E. coli* strains with a T7 polymerase-expressing vector. However, pRSETB-Peredox transformants formed red-tinted colonies on agar plates, and it was subsequently confirmed by fluorescence measurements, relative to the untransformed *E. coli* controls, that those cells expressed mCherry, confirming expression from the T7 operon (Figure 1). Consequently, it was confirmed by PCR for the T7 polymerase gene that neither of the *E. coli* strains was infected with the T7 phage (Supplementary Figure S2). It is therefore reasonable to assume that the mCherry expression is a result of ‘leaky’ expression from the T7 promoter and therefore this biosensor can be used in T7 polymerase negative *E. coli* strains without further genetic modification.

### 3.2. pRSETB-Peredox expression does not significantly affect the ciprofloxacin antibiotic-tolerant subpopulation

**Figure 2:**
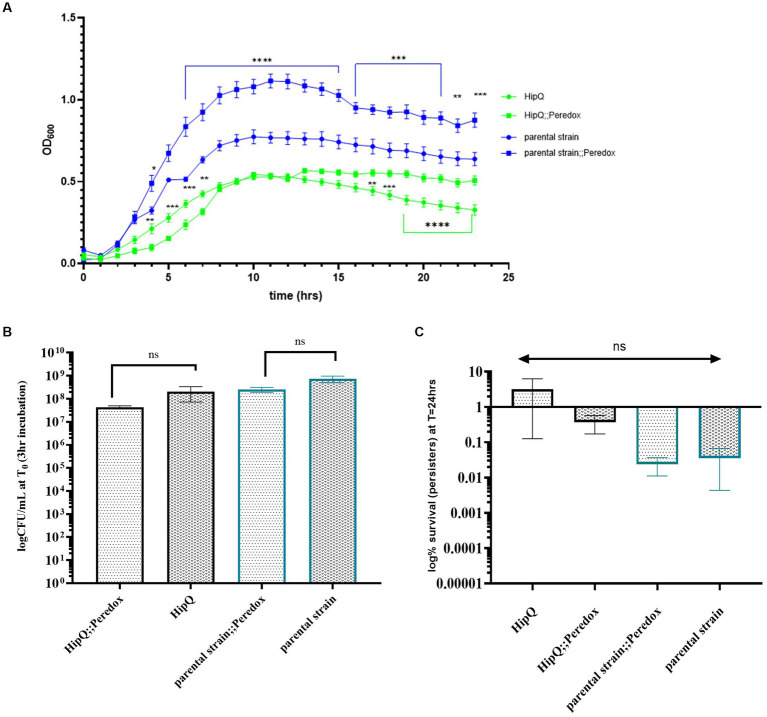
NADH biosensor (Peredox) expression does not significantly affect the growth phase at the starting point of the time kill assay, nor frequency of ciprofloxacin tolerant cells. **(A)** Optical density (OD_600_) growth curve of NADH biosensor expressing *E. coli* and their respective untransformed controls, n-3, error bars are SEM; ** *p* < 0.01, *** *p* < 0.001 and q < 0.05 by two-way ANOVA (strain vs. time) with Benjamini, Kreuger and Yekutieli false discovery control method. **(B)** Time-kill assay starting CFU/mL. **(C)** Ciprofloxacin persister frequency of untransformed and NADH biosensor (Peredox)-expressing *E. coli*. n-5 biological repeats for Peredox-expressing and n-3 biological repeats for untransformed strains, from 3 independent experiments; error bars are SEM. *p*. and q > 0.05 by one-way ANOVA with Benjamini, Kreuger and Yekuticli false discovery control method.

Once expression of the NADH biosensor was confirmed in *E. coli*, the next step was to investigate whether it affects antibiotic tolerance, possibly by slowing down cell growth and/or inducing the stress response due to the presence of protein aggregates (Schramm et al., 2020). Although growth rates, measured as optical density change over 24h, showed a significant difference between NADH biosensor-expressing cultures and their respective untransformed controls (Figure 2A), no statistically significant difference in CFU/mL was observed after at 3 h of growth (Figure 2B), the starting time point of the time-kill assays. Since CFU/mL measurements are considered the ‘gold-standard’ for growth assays, as they are not influenced by cell size and/or the presence of dead cells, both biosensor-expressing and untransformed cultures were assumed to be in the same growth phase at 3 h.

**Figure 3:**
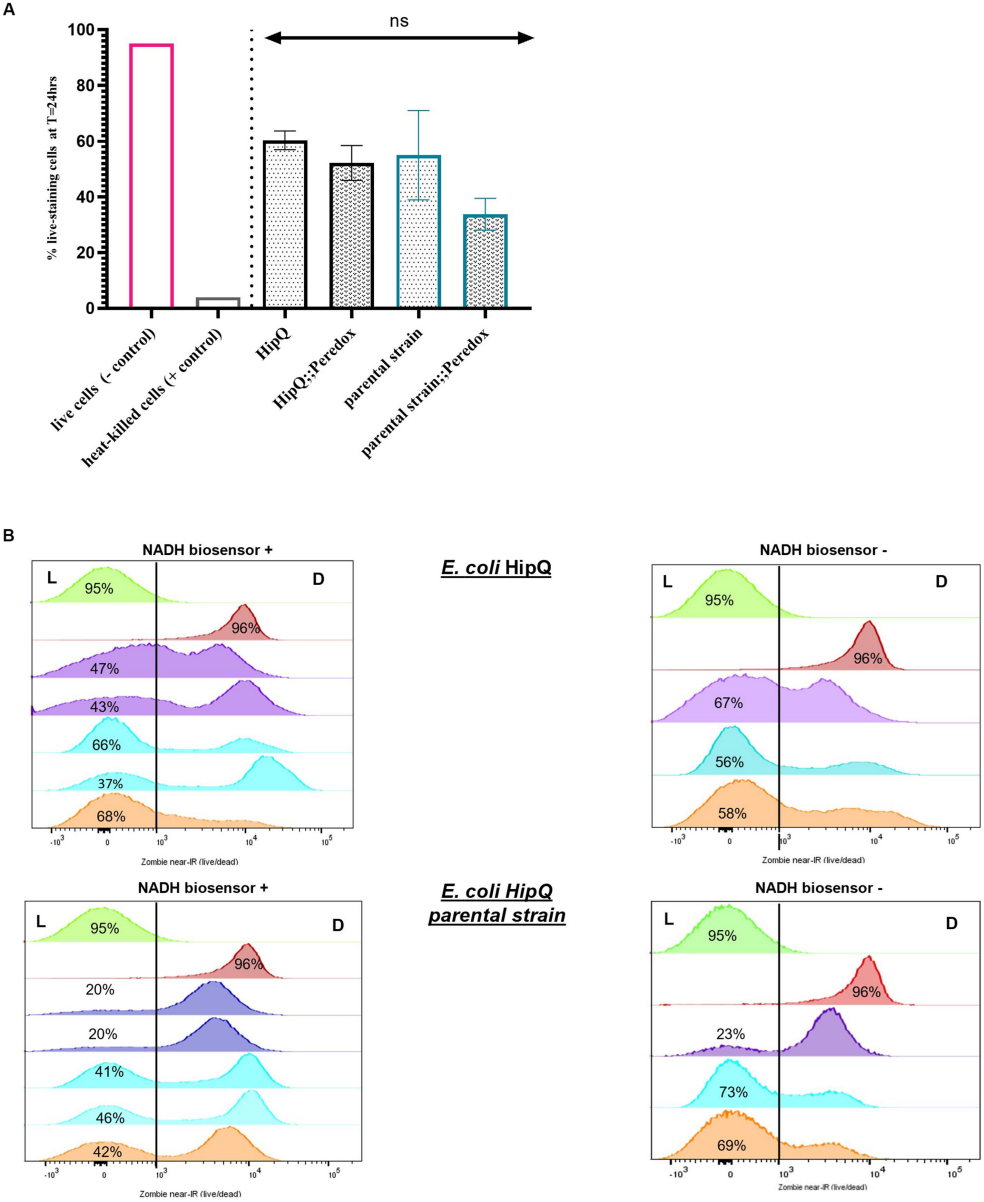
The antibiotic-tolerant subpopulation consists primarily of cells in the VBNC state. **(A)** % antibiotic-tolerant cells (persisters & VBNCs) following 24hr exposure to 25X MIC of ciprofloxacin. (n); n-5 for biosensor expressing and n-3 biological repeats for untransformed strains, from three independent experiments, 100,000 events/replicate; *p* > 0.05 by one-way ANOVA with and Benjamini, Kreuger and Yekutieli false discovery control method. **(B)** Zombie near-IR dye live/dead staining of *E. coli* HipQ and its parental strain following 24hr exposure to 25X MIC of ciprofloxacin. All panels: green - negative control (live cells) and red-positive control (heat-killed cells). Each histogram represents a biological replicate, 100,000 events/replicate were collected; matched replicates (biosensor + and from the same independent experiment) are colour coded. L, “live” population; D, “dead” population.

Next, ciprofloxacin persister frequencies of biosensor-expressing and untransformed cultures were compared. Ciprofloxacin was chosen as it is non-lytic (LeBel, 1988) so the biosensor would be retained in the cells following antibiotic exposure. Also, the HipQ strain is known to be highly persistent to fluoroquinolones (Wolfson et al., 1990). As can be seen in Figure 2C, there was no statistically significant difference in the % survival (persister subpopulation) of biosensor-expressing and untransformed cultures following 24 h of antibiotic exposure. There was also no statistically significant difference in the frequency of ciprofloxacin persisters between *E. coli* HipQ and its parental strain, however there was a high degree of variability between biological replicates. The difference in the frequency of persisters between HipQ and its parental strain was in line with previous observations, with *E. coli* HipQ generating 10–1,000 fold higher number of persister cells (Wolfson et al., 1990). It is possible that the non-lytic mechanism of action of ciprofloxacin could have resulted in a high frequency of damaged, VBNC-like cells whose regrowth was extremely sensitive to environmental conditions, resulting in increased variance in recorded frequencies of persister cells. This hypothesis was further validated by viability staining of antibiotic-exposed cultures (Figure 3), which demonstrated that a significant proportion of cells were alive following antibiotic exposure, although only a small minority of these cells regrew during our experimental timeframe.

**Figure 4:**
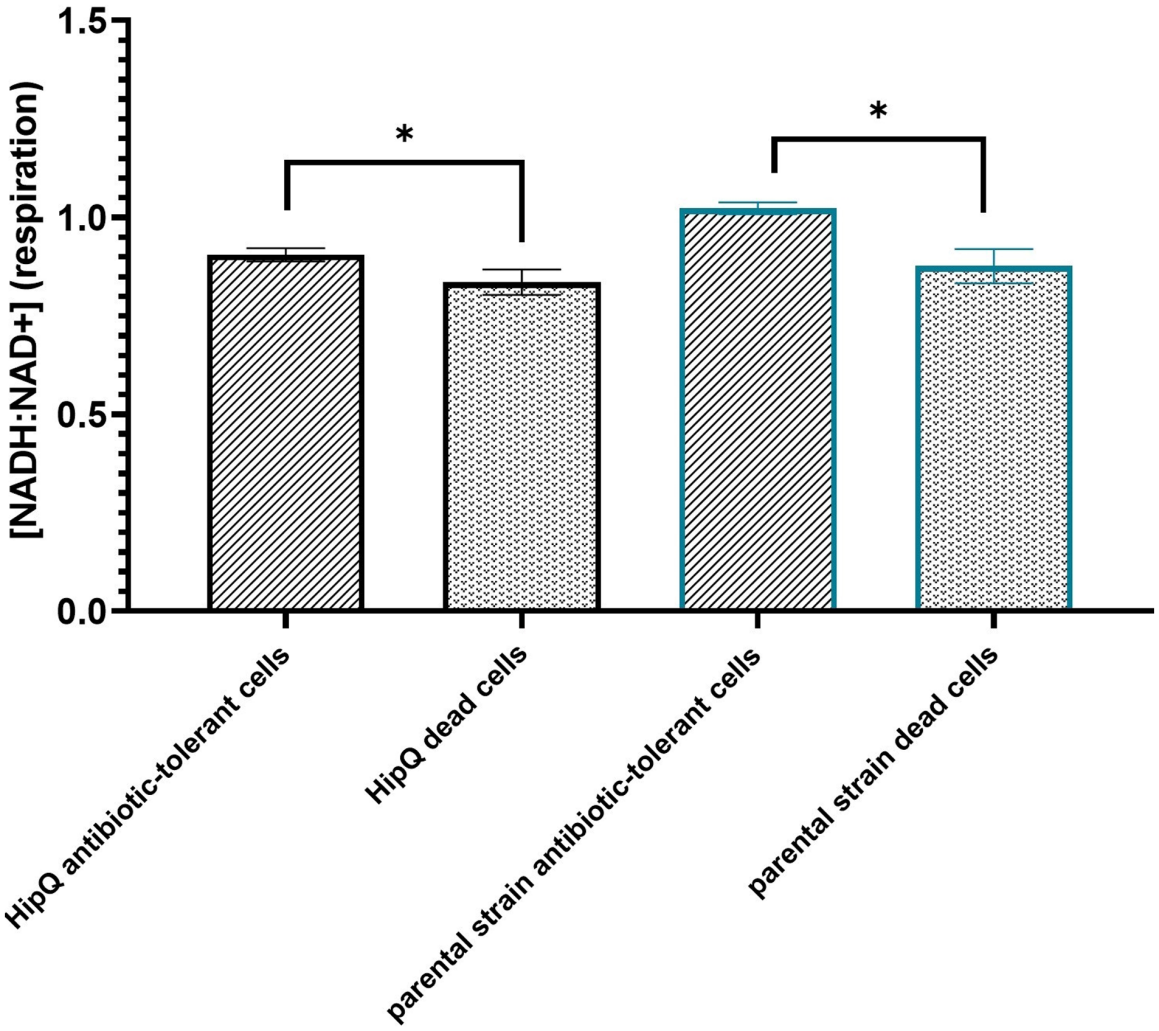
The antibiotic-tolerant subpopulation is metabolically active. Intracellular [NADH:NAD+] of antibiotic tolerant and dead cells following 24hr exposure to 25XMIC ciprofloxacin. n-5 biological repeats, from 3 independent experiments, 100,000 events per sample (n); error bars are SEM. *p < 0.05, *q* < 0.05 by RM ANOVA with Benjamini, Kreuger and Yekutieli false discovery control method.

Furthermore, there was no statistically significant difference in the average of total antibiotic-tolerant cells (persisters and VBNCs, or cells which stained as ‘live) following 24 h of ciprofloxacin exposure between biosensor-expressing and untransformed *E. coli* (Figure 3), or between HipQ and its parental strain. The frequency of live cells (persisters and VBNCs) was also several orders of magnitude higher than the frequency of persisters, demonstrating that following ciprofloxacin exposure the vast majority of the antibiotic-tolerant subpopulation is in the VBNC state. It is noted that the live/dead discrimination method was based on cellular membrane integrity and therefore could have led to an overestimation of ‘live’ cells, given the fact that ciprofloxacin is a non-lytic antibiotic. However, it was subsequently demonstrated, that ‘live’ cells undergo respiration at higher rates than ‘dead’ cells (Figure 4), investigated further in section 3.1.1 providing evidence that this subpopulation consists of metabolically active (i.e., living) cells.

**Figure 5:**
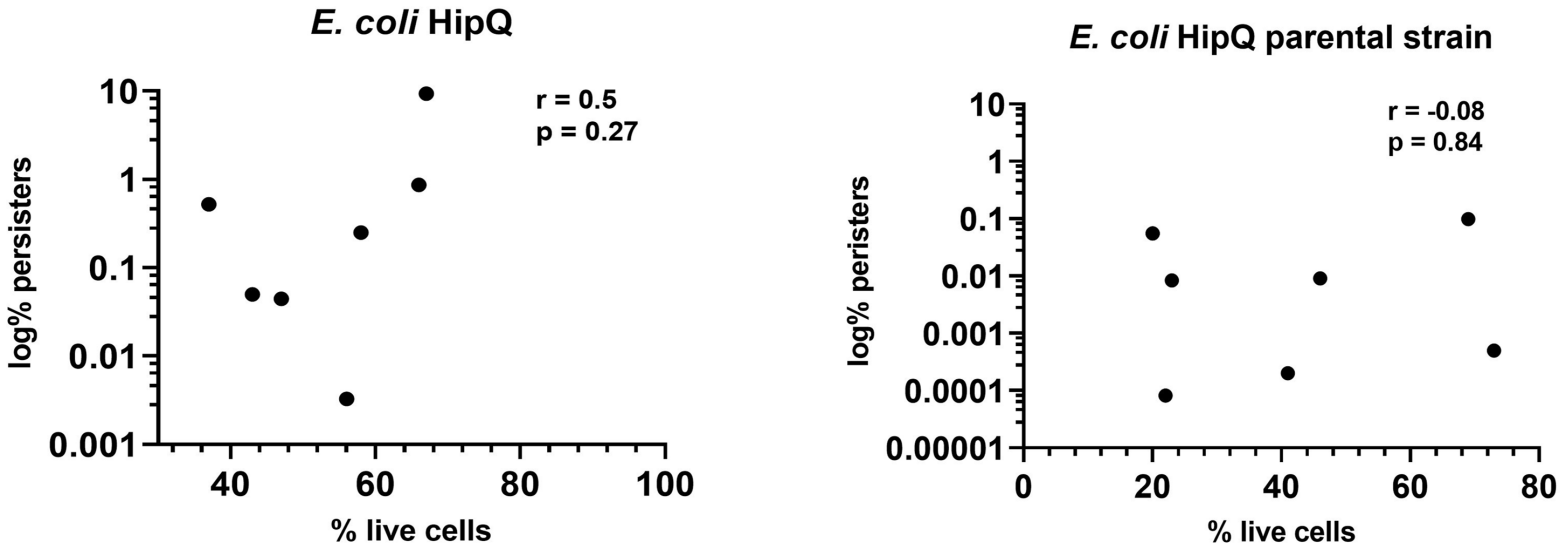
The frequency of antibiotic persisters does not correlate with the frequency of total antibiotic-tolerant cells. Spearman correlation (r) between % of antibiotic- tolerant cells (persisters &VBNCs) and % of persisters following 24hr exposure to 25XMIC ciprofloxacin. n-8 from 3 independent experiments; % of persisters is relative to the starting population and % of live cells is relative to the number of intact cells at t-24hrs of antibiotic exposure.

Interestingly, the frequency of persisters in a biological replicate did not correlate with the frequency of VBNCs, i.e., a higher % of live cells did not consistently equal to a higher number of persisters (Figure 5). This suggests that ciprofloxacin-related persister and VBNC state is potentially influenced by different mechanisms, or in other words that likelihood of regrowth is independent of likelihood of antibiotic exposure survival. This explanation is further supported by the finding that the proportion of total antibiotic-tolerant cells (persisters and VBNCs) is not significantly different between the highly-persistent HipQ strain and its parental counterpart after treatment with ciprofloxacin (Figure 3).

### 3.3. NADH/NAD+ homeostasis is disturbed in ciprofloxacin tolerant cells

In this assay exponentially growing cultures were exposed to 25XMIC of ciprofloxacin and cytosolic [NADH:NAD+], as well as viability of individual cells was measured with a flow cytometer. As mentioned previously (section 3.2), ciprofloxacin is non-lytic and therefore it was reasoned that the majority of ciprofloxacin-exposed cells will retain their protein content (including the Peredox biosensor), regardless of their viability status.

#### 3.3.1. Persisters and VBNCs are metabolically active following antibiotic exposure

First, the relative intracellular [NADH] was compared between ‘live’ and ‘dead’ subpopulations. As mentioned previously, the discriminating factor between live and dead cells was membrane integrity, and therefore there was a possibility of overestimating the ‘live’ subpopulation. However, it was hypothesized that live cells, whether persisters or VBNCs, would still undergo respiration and therefore should have a higher intracellular [NADH:NAD+] than dead cells (Pinzon et al., 2011; Ricciardi et al., 2014; Braissant et al., 2020). Since it was confirmed that there was no significant difference between autofluorescence of live and dead cells at T = 24 h (Supplementary Figure S3), these subpopulations were compared directly. When mCherry fluorescence intensity (plasmid copy number) was originally plotted against T-Sapphire fluorescence intensity, in 1 out of 3 independent experiments (*n* = 2) there was a subpopulation of ‘live’ cells which expressed mCherry at low levels, lower than the majority of ‘dead’ cells, yet higher than autofluorescence at this wavelength (Supplementary Figure S4). Consequently, the T-Sapphire fluorescence intensity of this subpopulation ([NADH:NAD+]) was comparable to autofluorescence of untransformed cultures. This subpopulation was not observed in the other two independent repeats of this assay (*n* = 3). It was possible that these cells could have been carrying a very low copy number of the NADH biosensor, since we have previously demonstrated that mCherry protein is stable up to 24 h post expression inhibition (data not shown). Alternatively, these cells could have been ‘dormant’ (not metabolically active) prior to ciprofloxacin exposure resulting in extremely low levels of biosensor expression, however if that was the case, they were functionally indistinct from dead cells. Due to its unique characteristics, this subpopulation was analyzed separately and is referred to below as the ‘zombie subpopulation’. Interestingly, no ‘zombie cells’ were observed in *E. coli* HipQ (Supplementary Figure S4).

As can be seen in Figure 4, there is a statistically significant difference between average intracellular [NADH:NAD+] between live (excluding the zombie subpopulation) and dead cells following exposure to ciprofloxacin. This indicates that VBNCs and persisters are actively undergoing respiration resulting in NAD+ reduction during glycolysis and the Krebs cycle (Massudi et al., 2012). Moreover, these observations validate membrane integrity in conjuction with metabolic activity measurements as a live/dead discrimination method for persisters and VBNCs.

#### 3.3.2. Ciprofloxacin-tolerant cells have a lower [NADH: NAD+] than the bulk population

**Figure 6:**
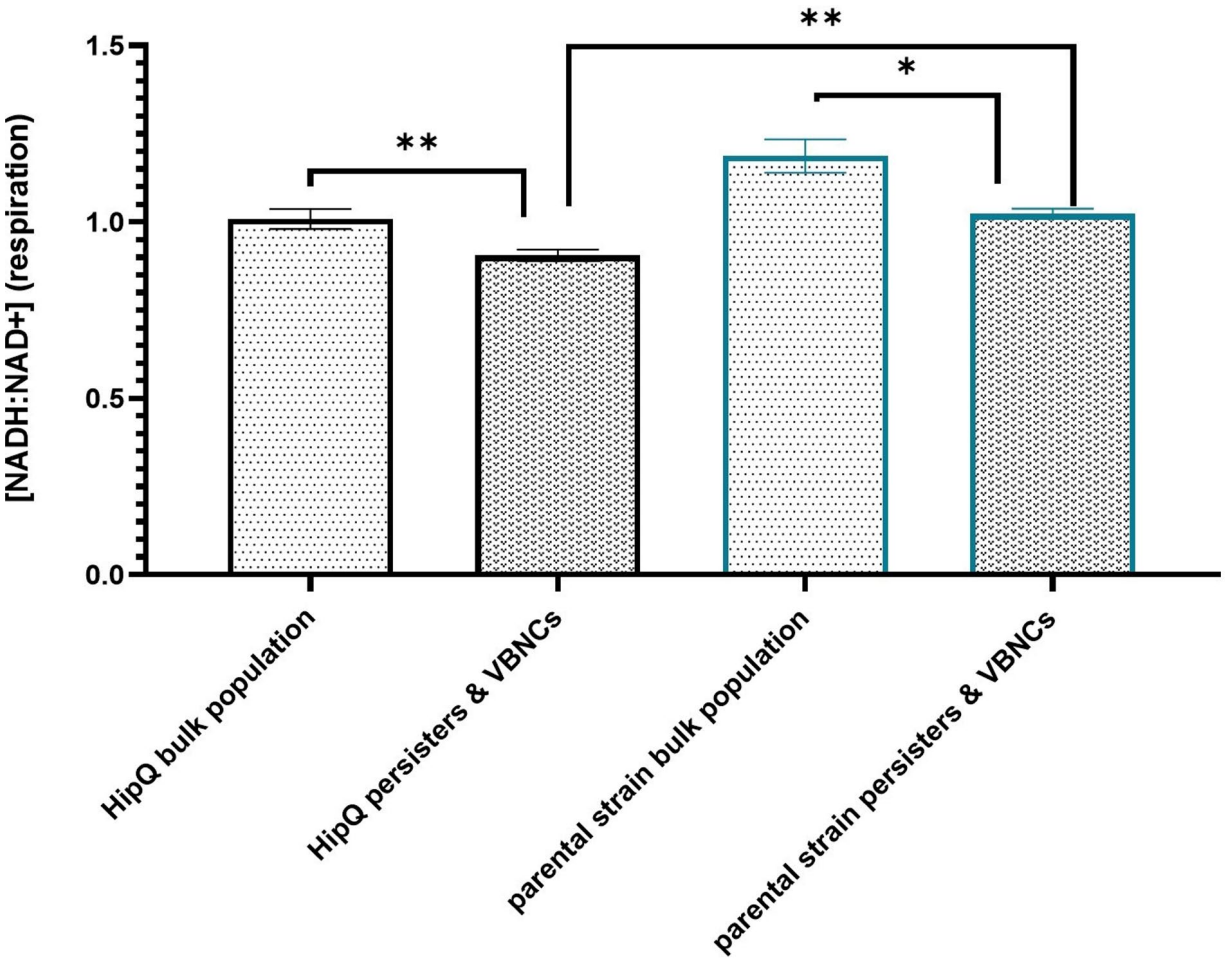
Antibiotic-tolerant cells undergo respiration at a significantly lower rate than the bulk population prior to antibiotic exposure. Intracellular [NADH:NAD+] of live cells prior (bulk population) and following (persisters & VBNCs) 24hr exposure to 25XMIC ofloxacin. n-5 biological replicates, from 3 independent experiments, 100,000 events per sample (n); error bars are SEM. * *p* < 0.05, *p* < 0.01, and q < 0.05 RM ANOVA with Benjamini, Kreuger and Yekutieli **. False discovery control method.

Next, [NADH:NAD+] was compared between antibiotic-tolerant cells (persisters & VBNCs excluding the zombie subpopulation) and the bulk population prior to antibiotic exposure. As can be seen in Figure 6, there is a statistically significant difference in intracellular [NADH:NAD+] between live cells at *T* = 24 h post antibiotic exposure and live cells prior to antibiotic addition, with antibiotic-exposed cells showing lower NADH:NAD+ ratio (corresponding to a lower respiration rate). The 405/520 nm (T-Sapphire) autofluorescence of live cells at *t* = 24 h vs. *t* = 0 h was not significantly different (Supplementary Figure S3), and therefore the respective fluorescence intensities presented in Figure 6 were compared directly.

Moreover, highly-persistent strain *E. coli* HipQ persisters and VBNCs showed a significantly lower [NADH:NAD+] than persisters and VBNCs of its parental strain despite no statistically significant difference at bulk population level prior to antibiotic exposure. This supports the hypothesis that perturbed NADH homeostasis relates to antibiotic tolerance. However, it is important to note that the ciprofloxacin-tolerant subpopulation was itself heterogeneous on a single cell level (Supplementary Figure S5) with a small fraction of antibiotic-tolerant cells having a higher NADH:NAD+ than the average for the bulk population prior to antibiotic exposure. It is also noted that in this assay the reported cytosolic [NADH:NAD+] of the antibiotic-tolerant cells is representative in majority of the VBNC subpopulation, since VBNC frequencies were several orders of magnitude higher that the frequencies of persister cells (Figures 2, 3).

## 4. Discussion

### 4.1. The vast majority of ciprofloxacin-tolerant cells are in the VBNC state

Previous research has demonstrated that ciprofloxacin exposure results in a high number of VBNC cells (up to 90% of the population) (Mason et al., 1995) which is consistent with observations presented in this article. This is likely a consequence of mechanism of action of this antibiotic since ciprofloxacin inhibits DNA gyrase, impairing DNA replication and repair without causing cell lysis (LeBel, 1988). However, VBNC cells have also been observed in response to lytic antibiotics, such as ampicillin, although at a much lower frequency (Mohiuddin et al., 2020b; Goode et al., 2021).

There has been an ongoing debate in the field whether the VBNC state is separate phenomenon to persistence, with some research groups reporting a distinct phenotypic state associated with VBNC cells (Bamford et al., 2017; Goode et al., 2021), and others suggesting that VBNCs are simply ‘aged’ persisters, which consequently display an extremely long lag phase (Ayrapetyan and Williams, 2018; Kim et al., 2018). Regardless of classification, VBNCs are a clinically relevant subpopulation and pathogen’s such as enteropathogenic *E. coli, Vibrio cholerae* (Senoh et al., 2012) and *Mycobacterium tuberculosis* (McCune et al., 1966) VBNC cells have been shown to be resuscitated in *in vivo* (or *in vivo* mimicking) conditions, which could contribute to infection recurrence. Therefore, better understanding of the VBNC state would aid in design of improved antibiotic treatment regimens, effective against heterogenous antibiotic-tolerant subpopulations which include both persisters and VBNCs.

It is important to note that the definition of ‘viability’ regarding the viable but non culturable state differs and can be based either on transcriptional/translational activity and membrane integrity (e.g., Goode et al., 2021) or on membrane integrity alone (e.g., Ayrapetyan and Williams, 2018; Kim et al., 2018). However, recent findings suggest that membrane integrity is not a sufficient determinant of viability and that there are cell-like particles, filled with protein aggregates or lacking cytosolic contents altogether, which maintain intact membranes (Song and Wood, 2021). The authors of the aforementioned findings suggest that these ‘shells’ could have been mistakenly classified as VBNCs in previous research. Therefore, in this article we based our live/dead cell discrimination on both membrane integrity and metabolic activity, demonstrating that ciprofloxacin VBNCs are not dead ‘shells’ as they actively undergo respiration. However, it is possible that the ‘zombie’ subpopulation we observed, which consisted of ‘live’ cells (i.e., cells with intact membranes) with a very low level of biosensor fluorescence were these cell-like particles described by Song and Wood (2021). Moreover, these observations further justify our reasoning of excluding the ‘zombie’ subpopulation from our analysis.

### 4.2. NADH homeostasis is dysregulated in antibiotic-tolerant cells

In this work, we sought to investigate the intracellular redox homeostasis of ciprofloxacin-tolerant cells. Previous work by Orman and colleagues has demonstrated, with the innovative use of flow cytometry combined with redox sensor green (RSG) staining, that in exponentially growing *E. coli* cultures, ofloxacin persisters were more likely to have a lower reductase activity than the bulk population (Amato et al., 2013). We validated these results with a closely related quinolone, ciprofloxacin. However, we note that reductase activity measured by Orman et al. is not specific to [NAD(H)], as there are NAD(H) independent redox reactions occurring within cells, such as NADP(H) or ATP mediated reactions (Gowda, 2018). To the best of our knowledge the enzyme(s) involved in RSG dye redox reaction, used by the authors of the aforementioned study, are not specified. Here we demonstrated that subpopulation tolerance to ciprofloxacin in *E. coli* is related specifically to a lower intracellular [NADH] and subsequently, downregulated cellular respiration (Pinzon et al., 2011; Ricciardi et al., 2014; Braissant et al., 2020). Respiration-inducing drugs have been previously reported to be active against ciprofloxacin persisters (Marques et al., 2014), and our work suggests that those could also be effective against VBNCs. However, we also found that cells that survive prolonged antibiotic exposure consisted of two subpopulations: those whose [NADH:NAD+] was lower than the bulk population prior to antibiotic exposure and a smaller subpopulation with a higher NADH:NAD+ than the bulk population. This is further evidence that the antibiotic-tolerant subpopulation is itself heterogenous and suggests a combination therapy approach might be necessary for its eradication. It is noted that coenzyme flavin adenine nucleotide (FAD) has a similar fluorescence spectrum to T-Sapphire. Cytosolic FAD has previously been shown to increase in stressful environmental conditions, such as antibiotic exposure (Surre et al., 2018). Although in this work we did not find a significant increase of autofluorescence between the bulk population and antibiotic-tolerant cells on batch level, it is possible that on a single-cell level antibiotic-tolerant cells which displayed a high level of fluorescence produced a higher amount of FAD, rather than NADH.

### 4.3. NADH homeostasis is affected in the highly-persistent strain *Escherichia coli* HipQ

It has been previously shown that *E. coli* HipQ generates 10–1000 fold more ofloxacin persisters than its parental strain (Wolfson et al., 1990). This agrees with ciprofloxacin persister cell frequencies presented in this work, since ofloxacin is a closely related quinolone. Moreover, we demonstrated that *E. coli* HipQ antibiotic-tolerant cells have a lower [NADH:NAD+] than those of its parental strain. This suggested that respiration occurs at a lower rate in these cells. *E. coli* HipQ has previously been shown to generate an increased number of stochastic persisters, i.e., cells at the ‘tail-end’ of growth/division rate distribution (Balaban et al., 2004; Hingley-Wilson et al., 2020). Our observations point towards a link between stochastic persistence and cellular respiration, with cells at the ‘tail-end’ of the respiration rate distribution being more likely to survive antibiotic exposure, however this hypothesis requires further experimental validation.

### 4.4. Conclusion, limitations, and future work

This study presents a framework for the use of NADH biosensor Peredox in investigations of NAD(H) homeostasis of antibiotic-tolerant and antibiotic-susceptible bacterial cells.

In this work we demonstrated that the majority of *E. coli* ciprofloxacin-tolerant cells are in the VBNC state. These observations could be easily expanded on in the future work to other non-lytic antibiotics and/or bacterial species, following the method we established and validated here. Furthermore, we evaluated dysregulated NADH homeostasis as a potential marker for antibiotic tolerance and showed that the antibiotic tolerant subpopulation differs significantly from the bulk population in their cytosolic [NADH:NAD+]. We note that the biggest limitation of our study is that we were not able to determine whether this dysregulation occurs as cause or effect of antibiotic tolerance (in other words, whether antibiotic-tolerant cells showed this NAD(H) homeostasis dysregulation prior to antibiotic exposure). To expand on the observations presented here, we suggest employing fluorescence-activated cell sorting (FACS), in order to sort populations based on [NADH:NAD+] prior to antibiotic exposure, which would allow to answer the aforementioned research question, and subsequently validate whether [NADH:NAD+] can be used as antibiotic tolerance marker. Furthermore, to increase sensitivity of this system, higher level of Peredox expression could be obtained by inducing T7 promoter-dependent expression. However, the potential effect of high amounts of recombinant protein on cellular metabolic state (e.g., through ribosomal sequestration or formation of protein aggregates), and subsequently antibiotic tolerance, would need to be evaluated first.

## Data availability statement

The datasets presented in this study can be found in online repositories. The names of the repository/repositories and accession number(s) can be found at: Flow Repository: http://flowrepository.org/id/FR-FCM-Z64Q.

## Author contributions

JU: study design, data collection and analysis, and draft manuscript preparation. FH, JM, MS-S, and SH-W: supervision and critical revision of data and manuscript. All authors contributed to the article and approved the submitted version.

## Funding

This work was funded by the MRC/AMED grant MR/T028998/1.

## Conflict of interest

The authors declare that the research was conducted in the absence of any commercial or financial relationships that could be construed as a potential conflict of interest.

## Publisher’s note

All claims expressed in this article are solely those of the authors and do not necessarily represent those of their affiliated organizations, or those of the publisher, the editors and the reviewers. Any product that may be evaluated in this article, or claim that may be made by its manufacturer, is not guaranteed or endorsed by the publisher.
