# Supplementary material for: Dysregulated NAD(H) homeostasis associated with ciprofloxacin tolerance in Escherichia coli investigated on a single-cell level with the Peredox [NADH:NAD+] biosensor
Source: Front Microbiol. 2023 Jun 21;14:1191968. doi: 10.3389/fmicb.2023.1191968 (PMC10321300; doi:10.3389/fmicb.2023.1191968)
Supplement: Supplementary file 1 [file Data_Sheet_1.pdf]

Supplementary Material

1. **Peredox biosensor plasmid map**

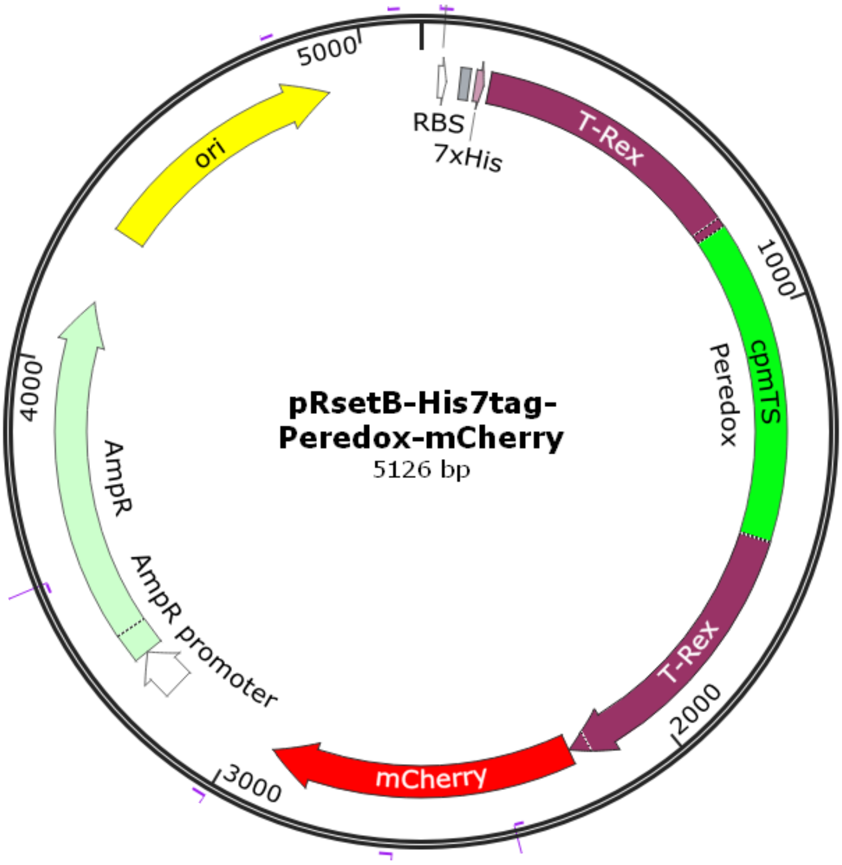

Figure S1. pRSETB-Peredox (Addgene plasmid #32382).

2. **T7 polymerase gene PCR**

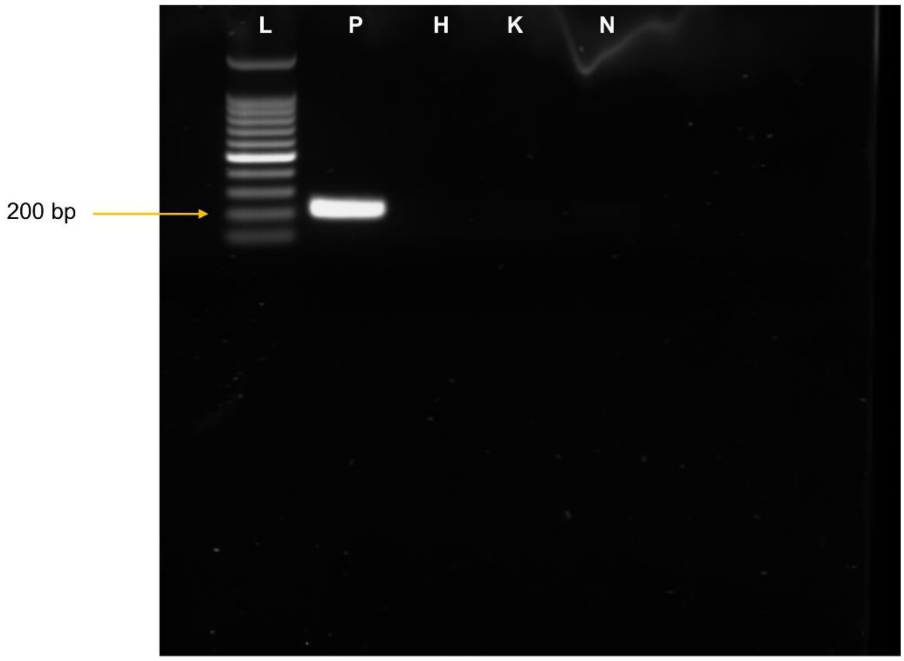

Figure S2. *E. coli* HipQ and its parental strain are not infected with a functional T7 phage. 2% agarose gel electrophoresis of T7 polymerase gene detection PCR. From left to right: L - 100bp DNA ladder (Promega), P - positive control (*E. coli* BL21(DE3)), H - *E. coli* HipQ, K - *E. coli* HipQ parental strain, N – no template control; expected band size was 228bp.

3. 405/520nm (T-Sapphire wavelength) autofluorescence of *E. coli*

A.

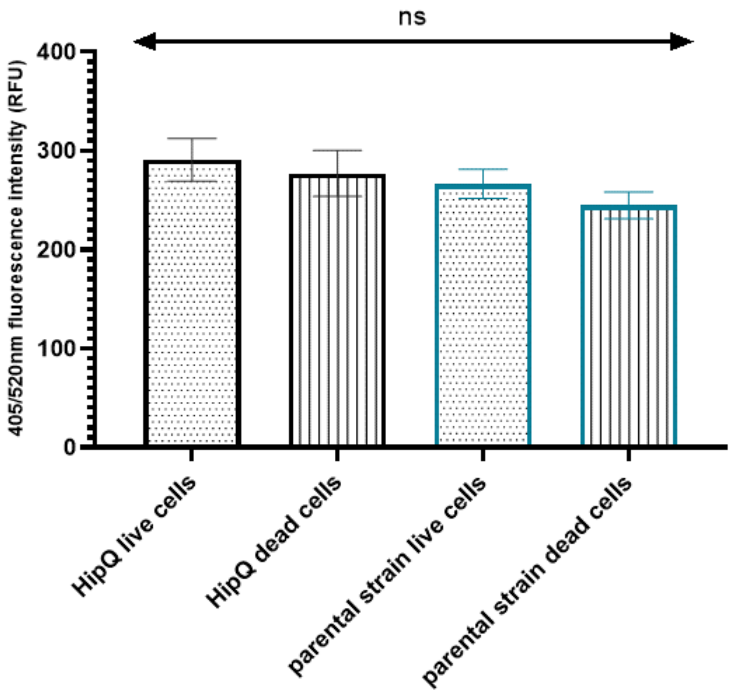

B.

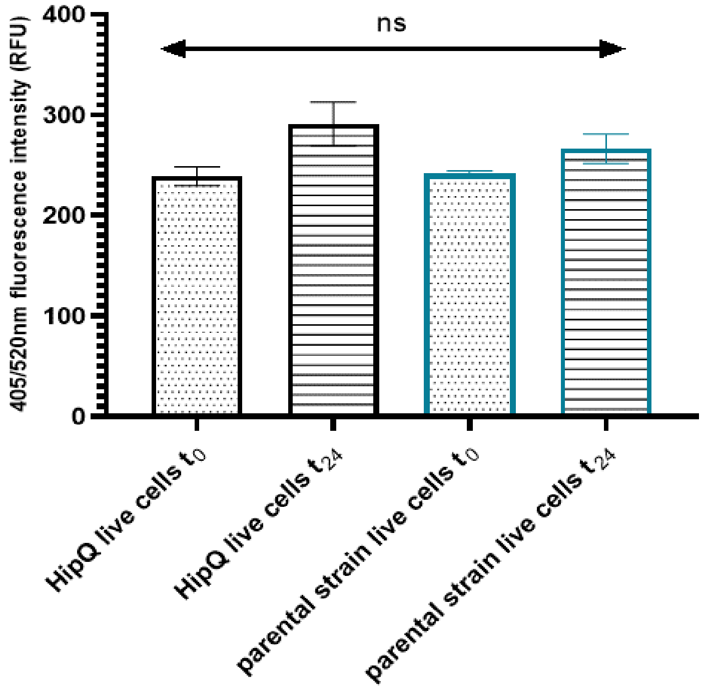

Figure S3. Green (T-Sapphire wavelength) autofluorescence of *E. coli* HipQ is not significantly different than respective autofluorescence of its parental strain, and is not significantly affected by ciprofloxacin exposure or cell viability. 405/520nm autofluorescence intensity of A. live

(persisters and VBNCs) and dead and B. live at T=0hrs and T=24hrs *E. coli* cells following 24hr exposure to 25X MIC of ciprofloxacin. n=3 from three independent experiments, error bars are SEM; 100 000 events/replicate were collected; $p > 0.05$ by repeated measures ANOVA with Geiser-Greenhouse correction and with Benjamini, Kreuger and Yekutieli false discovery control method, values were matched within experiment.

4. Cytosolic [NADH:NAD+] measured on single-cell level

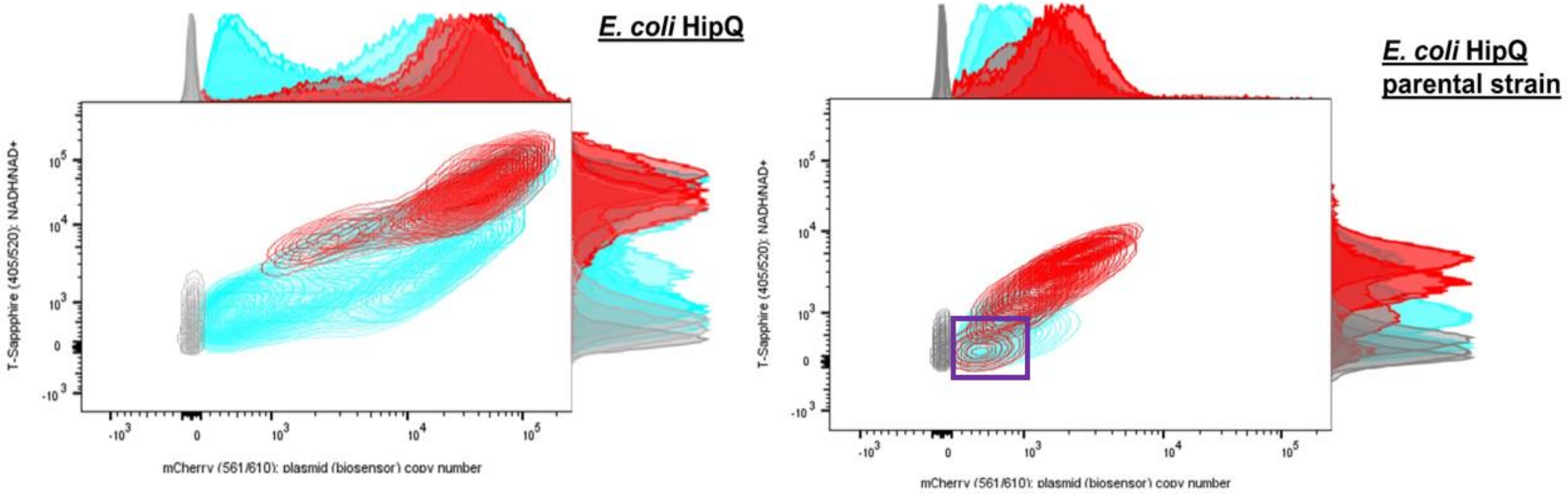

Figure S4. The ciprofloxacin tolerant subpopulation actively undergoes respiration. Intracellular NADH:NAD+ of *E. coli* cells 24hrs post exposure to 25X MIC of ciprofloxacin. In blue are dead cells, in red are live cells, and in grey is autofluorescence (cultures not expressing the NADH biosensor exposed to the same conditions); ‘zombie’ subpopulation is highlighted in purple. Higher NADH:NAD+ ratio corresponds to higher rate of cellular respiration. n=5 from 3 independent experiments; 1000k events per sample were collected.

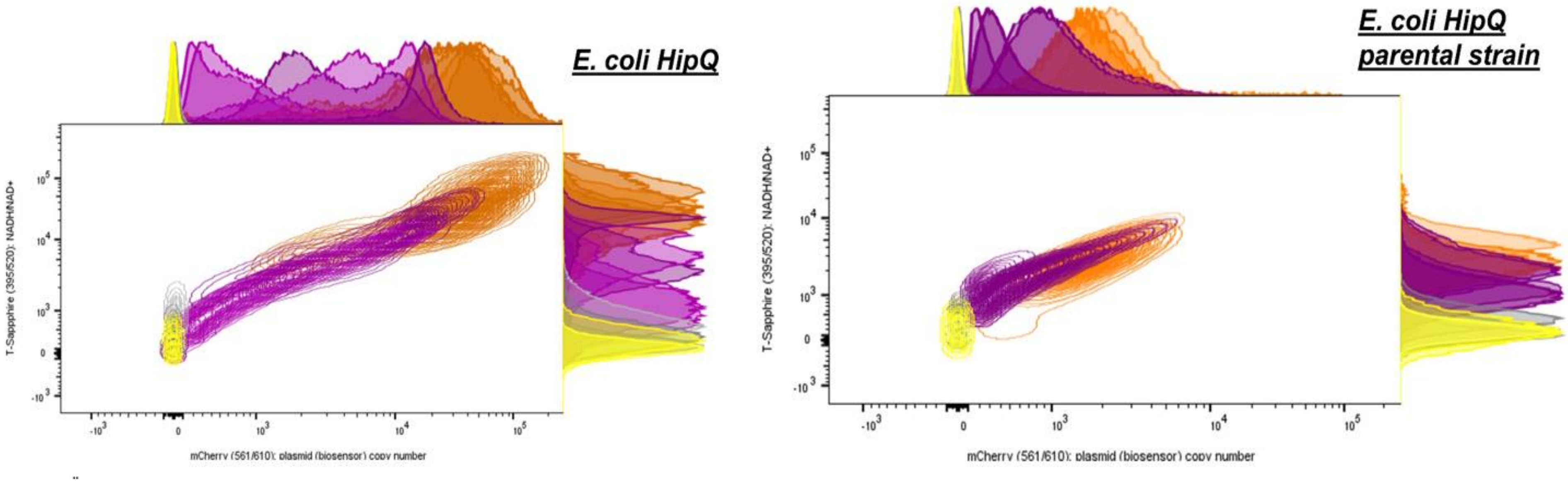

Figure S5. On a single cell level, heterogeneity in respiration rates can be observed within the antibiotic-tolerant subpopulation. Intracellular NADH:NAD+ of live *E. coli* prior (purple) and post (orange, excluding the zombie subpopulation) 24hrs exposure to 25X MIC of ciprofloxacin. In yellow is autofluorescence at T=0hrs and in grey is autofluorescence at T=24hrs of antibiotic exposure. Higher NADH:NAD+ ratio corresponds to higher rate of cellular respiration. n=5 from 3 independent experiments; 1000k events per sample were collected.
